# Supplementary material for: A brain network that supports consensus-seeking and conflict-resolving of college couples’ shopping interaction
Source: Sci Rep. 2020 Oct 19;10:17601. doi: 10.1038/s41598-020-74699-1 (PMC7573624; doi:10.1038/s41598-020-74699-1)
Supplement: Supplementary file 1 — Supplementary Information. [file 41598_2020_74699_MOESM1_ESM.docx]

**A brain network that supports consensus-seeking and conflict-resolving of college couples’ shopping interaction**

**HanShin Jo^1,2^, Chiu-Yueh Chen^2,3^, Der-Yow Chen^2,5^, Ming-Hung Weng^4^, Chun-Chia Kung*^2,5^**

^1^ Inst. of Medical Informatics, National Cheng Kung University (NCKU), Tainan, Taiwan
^2^ Dept. of Psychology, NCKU, Tainan, Taiwan

^3^ KU Leuven, Belgium

^4^ Dept. of Economics, NCKU, Tainan, Taiwan

^5^ Mind Research and Imaging (MRI) Center, Tainan, Taiwan

Corresponding author e-mail: [cckung@kunglab-nckupsy.org](mailto:cckung@kunglab-nckupsy.org)

**Supplementary information**

|  | Estimate | SE | tStat | pValue |  | Estimate | SE | tStat | pValue |
| --- | --- | --- | --- | --- | --- | --- | --- | --- | --- |
| Alone | ________ | ________ | ________ | ________ | Together | ________ | ________ | ________ | ________ |
| (Intercept) | -2.4475 | 0.87615 | -2.7934 | 0.0052 | (Intercept) | -3.7678 | 0.66254 | -5.6869 | 0.0000 |
| self_rank | 2.4874 | 0.13897 | 17.898 | 0.0000 | self_rank | 2.801 | 0.12679 | 22.092 | 0.0000 |
| price | -0.00035493 | 6.35E-05 | -5.5912 | 0.0000 | ranking_diff | -0.97043 | 0.18667 | -5.1986 | 0.0000 |
| sex_female | -0.031045 | 0.17426 | -0.17815 | 0.85861 | price | -8.55E-05 | 2.24E-05 | -3.8175 | 0.0001 |
| age | -0.18324 | 0.036711 | -4.9913 | 0.0000 | sex_female | -0.13655 | 0.13176 | -1.0364 | 0.30001 |
|  |  |  |  |  | age | -0.16101 | 0.027318 | -5.894 | 0.0000 |
|  |  |  |  |  | self_rank:diff | 0.01866 | 0.065315 | 0.28569 | 0.77511 |

[Supplementary table S1. Behavioral logistic regression] The logistic regression model estimates buying decision (buy/not buy) in alone and together condition, revealing that own preference, price, and age are factors that significantly predict one’s behaviour in both “shop alone” and “shop together” conditions. Additionally, in “shop together” trials, the differences between the preferences of own and other showed significantly negative correlation, indicating the modulatory role of significant others’ preferences in one’s own shopping decisions.

| Cluster table of map: "Together > Alone" | | | | |
| --- | --- | --- | --- | --- |
| x, y, z | k | max | mean | tdclient |
| ----------------------------------------------------------- | | | | |
| -12, 56, 27 | 959 | 7.040407 | 3.553929 | LH Superior Frontal Gyrus (Brodmann area 9) (-12; 58; 27) [d=2.0mm] |
| -43, -58, -8 | 1169 | 6.532799 | 3.44449 | LH Fusiform Gyrus (Brodmann area 37) (-41; -60;-11) [d=4.1mm] |
| 33, 16, -11 | 109 | 6.304917 | 3.499791 | RH Inferior Frontal Gyrus (Brodmann area 47) |
| 12, -85, 6 | 463 | 6.013853 | 3.416915 | RH Cuneus (Brodmann area 17) |
| -3, -50, 25 | 205 | 5.886473 | 3.493299 | LH Posterior Cingulate (Brodmann area 23) |
| -39, 17, -10 | 309 | 5.851212 | 3.425125 | LH Inferior Frontal Gyrus (Brodmann area 47) |
| -49, 7, 41 | 88 | 5.274084 | 3.258311 | LH Middle Frontal Gyrus (Brodmann area 8) (-50; 7; 41) [d=1.0mm] |
| 53, -61, 22 | 283 | 5.165014 | 3.290112 | RH Middle Temporal Gyrus (Brodmann area 39) |
| 55, -1, -15 | 88 | 5.131149 | 3.368571 | RH Middle Temporal Gyrus (Brodmann area 21) ( 57; -3;-15) [d=2.8mm] |
| 19, 5, 11 | 97 | 4.97557 | 3.379958 | RH Lentiform Nucleus (Putamen) ( 20; 4; 11) [d=1.4mm] |
| 30, -80, 8 | 71 | 4.644316 | 3.138276 | RH Middle Occipital Gyrus (Brodmann area 19) ( 32; -83; 8) [d=3.6mm] |
| 50, 2, 39 | 76 | 4.283547 | 3.184655 | RH Middle Frontal Gyrus (Brodmann area 6) |
|  | | | | |
| Cluster table of map: "Congruent > Incongruent" | | | | |
| x, y, z | k | max | mean | tdclient |
| ----------------------------------------------------------- | | | | |
| 9, -78, 27 | 1309 | 4.855926 | 2.582847 | RH Cuneus (Brodmann area 18) |
| 28, 30, 12 | 200 | 4.706691 | 2.641261 | RH Insula (Brodmann area 13) ( 31; 22; 12) [d=8.5mm] |
| -31, 15, 31 | 167 | 4.503531 | 2.611587 | LH Middle Frontal Gyrus (Brodmann area 9) (-35; 15; 31) [d=4.0mm] |
| -61, -30, 15 | 184 | 4.14919 | 2.558136 | LH Superior Temporal Gyrus (Brodmann area 42) (-61; -28; 15) [d=2.0mm] |
| 37, -20, 8 | 142 | 3.892134 | 2.526385 | RH Insula (Brodmann area 13) ( 38; -20; 8) [d=1.0mm] |
| 39, 7, 29 | 213 | -5.91709 | -2.715866 | RH Inferior Frontal Gyrus (Brodmann area 9) ( 39; 5; 29) [d=2.0mm] |
| 0, 13, 42 | 212 | -4.760147 | -2.554845 | LH Medial Frontal Gyrus (Brodmann area 6) ( -1; 13; 43) [d=1.4mm] |
| -45, 13, 26 | 117 | -4.220338 | -2.646572 | LH Middle Frontal Gyrus (Brodmann area 9) |
| 31, -68, -6 | 113 | -3.504229 | -2.371885 | RH Fusiform Gyrus (Brodmann area 19) ( 31; -68; -7) [d=1.0mm] |

[Supplementary table S2. GLM contrast cluster table] The GLM contrast cluster table in the two contrasts: “Together > Alone” and “Congruent > Incongruent”. All brain coordinates are reported in the standard MNI space, and the contrasts are thresholded at p < 0.01 (Top) and p < 0.05 (Bottom). The cluster threshold was determined by AlphaSim in Neuroelf, using smoothness parameters estimated from the residuals of the statistical map.


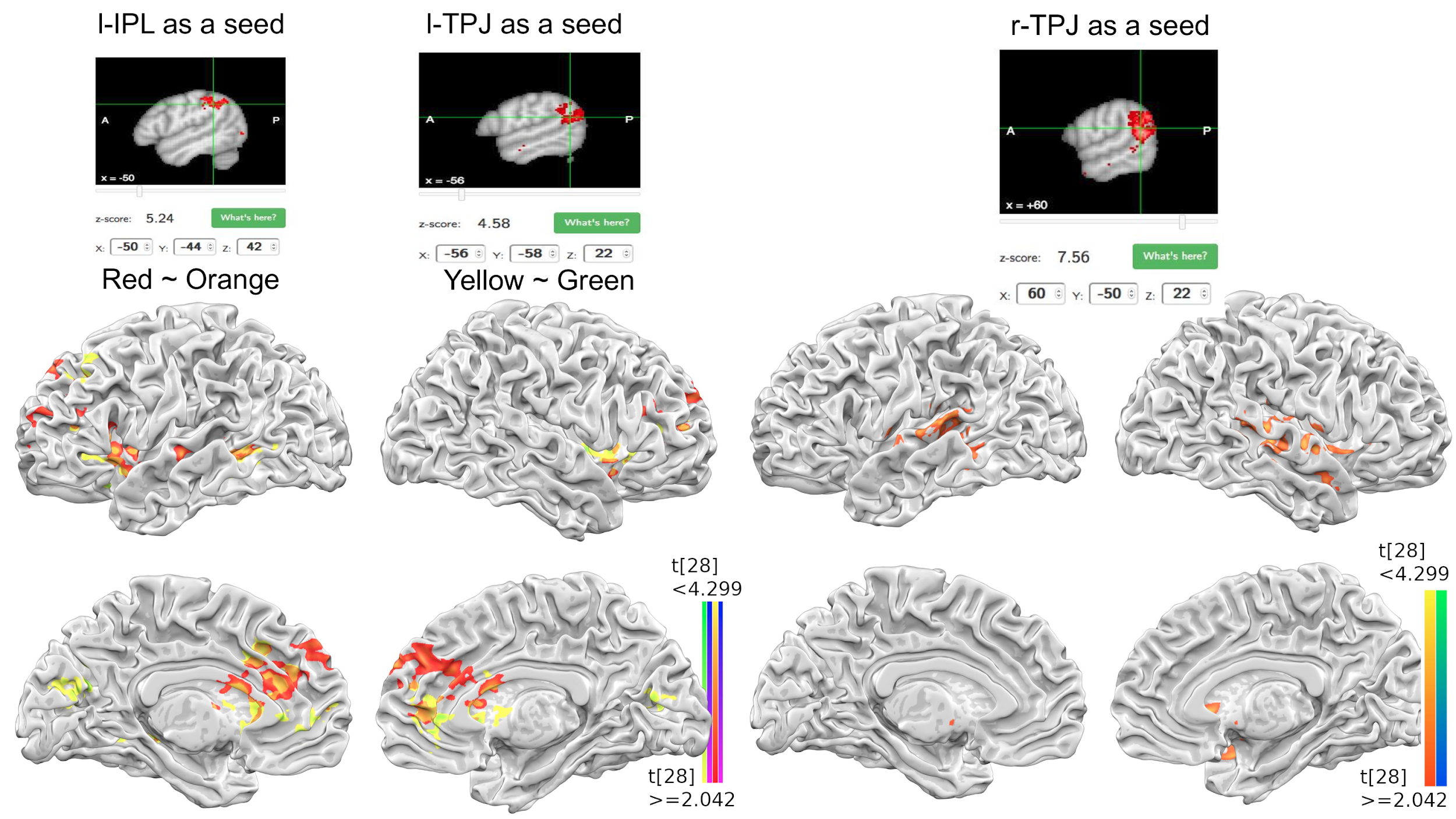


[Supplementary figure S1. PPI results of three different seed regions shown in separate] The similar functional connectivity between l-IPL and l-TPJ seed were overlaid (left), while the distinct functional connectivity map was shown with r-TPJ seed (right). All brain maps are thresholded at p < 0.05, FWE corrected.
